# Do we have to reconsider the evolutionary emergence of myelin?

**DOI:** 10.3389/fncel.2013.00217

**Published:** 2013-11-15

**Authors:** Hauke B. Werner

**Affiliations:** Department of Neurogenetics, Max Planck Institute of Experimental MedicineGöttingen, Germany

**Keywords:** myelination, saltatory impulse propagation, oligodendrocyte, Schwann cell, genomics, vertebrate evolution, lamprey, myelin basic protein (MBP)

A recent publication (Smith et al., 2013) reported the genome sequence of the sea lamprey (*Petromyzon marinus*). This dataset allows the exploration of early vertebrate evolution because the lamprey lineage split from that of other vertebrates before the emergence of hinged jaws, the defining feature of jawed vertebrates (gnathostomata, ranging from sharks to humans). Additionally, lamprey axons are not ensheathed by myelin (Bullock et al., 1984), a glial specialization that facilitates the rapid propagation of nerve signals (Hartline and Colman, 2007). It is therefore thought that myelin evolved at about the same time as hinged jaws in now-extinct placoderms, the most ancient group of jawed vertebrates (Zalc et al., 2008). Now, Smith et al. have examined the lamprey genome (Smith et al., 2013) to determine whether or not myelin genes evolutionarily pre-dated jawed vertebrates. Several myelin genes were indeed identified. For that reason, the authors propose the intriguing possibility that myelinating cells may have existed already in non-jawed vertebrates and were subsequently lost in the lamprey lineage. This hypothesis is in disagreement with all previous molecular and morphological analyses.

The evolution of myelin basic protein (MBP) is particularly relevant because MBP is an abundant structural myelin constituent essential for myelination in the central nervous system (CNS). The fact that MBP had not been traced in species more ancient than gnathostomata (Gould et al., 2005; Nawaz et al., 2013) founded the concept that myelin and MBP emerged at about the same time, probably interrelated. As one key observation, Smith et al. report a segment in the lamprey genome that—if translated into protein—would be 86% identical over 22 amino acids to a fragment of MBP (Smith et al., 2013). Smith et al. thus propose that MBP and myelinating cells may have evolved before jawed vertebrates.

**Figure 1:**
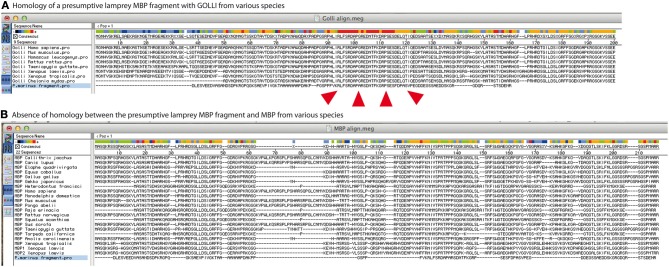
**A possible ortholog of gene-of-the-oligodendrocyte-lineage (GOLLI) protein, not myelin basic protein (MBP), was identified in the sea lamprey genome. (A)** GOLLI and **(B)** MBP protein sequences of the indicated species were retrieved from the NCBI database and aligned with the presumptive MBP fragment identified in the sea lamprey genome by Smith et al. (2013). The software Megalign from the Lasergene package was used with standard slow-accurate parameters of ClustalW and protein weight matrix Gonnet25O. Note that the sea lamprey fragment is highly homolog to GOLLI over a stretch of more than 20 amino acids as indicated by the red coloring in the consensus sequence [marked by the red arrowheads in **(A)**]. In contrast, no considerable similarity with MBP was identified **(B)**.

We note however that the identified fragment is not homolog to MBP. Instead it is homolog to the protein product of the *gene-of-the-oligodendrocyte-lineage* (GOLLI) (Figure 1). The function of GOLLI is not well understood. However, unlike MBP, GOLLI is not basic, not incorporated into the myelin sheath, and not essential for myelination (Jacobs et al., 2005; Nawaz et al., 2013). MBP and GOLLI are thus unrelated by sequence and distinct by function. The most likely explanation for the annotation of the *golli* fragment as *mbp* is that both occur in the same transcription unit in a range of species (Pribyl et al., 1993; Saavedra et al., 1993; Nawaz et al., 2013). The corresponding mRNAs, many of which encode both GOLLI and MBP, are commonly designated as “MBP” in databases, which also led to the annotation of ESTs as *mbp* even if they comprise only *golli*. This indicates that the automated alignment and annotation of nucleotide sequences can introduce systematic errors of designation into the databases. Together, a segment homolog to *golli* exists in the lamprey genome, but evidence of *mbp* is lacking.

Other presumed myelin proteins traced in the lamprey genome are not functionally related to myelin at all, despite an equivalent gene ontology (GO) term. For example, MYT1L (myelin transcription factor-1-like), a neuronal transcription factor (Kim et al., 1997), and MAL2 (myelin and lymphocyte protein-2), a constituent of synaptic vesicles and hepatocytes (De Marco et al., 2002; Gronborg et al., 2010), bear the term “myelin” in their name simply because of their homology with the founding members of their respective protein families. This suggests that the predictive value of GO terms must be viewed carefully.

On the other hand, the myelin proteins CNP (cyclic nucleotide phosphodiesterase), PMP22 (peripheral myelin protein of 22 kDa), MAL (myelin and lymphocyte protein), and PLP (proteolipid protein) were previously noted to be evolutionarily older than vertebrates (Mazumder et al., 2002; Gould et al., 2005; Mobius et al., 2008); MPZ (myelin protein zero, P0) is a member of the superfamily of cell adhesion molecules with immunoglobulin-like domains (Ig-CAM) that exist not only in vertebrates but also in invertebrates. These proteins were thus recruited in ancient vertebrates as myelin constituents from other cellular functions. Accordingly, the identification of corresponding gene segments in lamprey does not help in the identification of the evolutionary emergence of myelinating cells. It is noteworthy that none of these myelin genes is essential for myelination in mice—in contrast to MBP, which is required for membrane growth and compaction of CNS myelin.

Taken together, the lamprey genome does not provide reason to consider that myelin may have evolved in non-jawed vertebrates. More generally, conclusions from genomic datasets on cellular structures come with the danger of misinterpretations if not carefully considered in conjunction with morphological analyses (Bullock et al., 1984; Schweigreiter et al., 2006; Zalc et al., 2008). Indeed, absence of evidence for MBP in lamprey rather supports the concept that the emergence of myelin was coupled to the emergence of MBP. This innovation of myelin in ancient jawed vertebrates has sped up nerve conduction velocity more than 20-fold, a prerequisite for the evolution of large-bodied fish with a predatory lifestyle. Without myelin, jawed vertebrates as we know them, including us humans, could not have evolved.
